# Uncooled, broadband terahertz bolometers using SOI MEMS beam resonators with piezoresistive readout

**DOI:** 10.1038/s41378-025-00996-2

**Published:** 2025-07-07

**Authors:** Ya Zhang, Kazuki Ebata, Mirai Iimori, Qian Liu, Zihao Zhao, Ryotaro Takeuchi, Hua Li, Kazusuke Maenaka, Kazuhiko Hirakawa

**Affiliations:** 1 Institute of Engineering, Tokyo University of Agriculture and Technology, 2-24-16, Koganei-shi, Tokyo 184-8588 Japan; 2 Shanghai Institute of Microsystem and Information Technology, Chinese Academy of Sciences, Shanghai, 200050 China; 3 Department of Electrical Engineering and Computer Sciences, University of Hyogo, Himeji, Japan; 4 Institute of Industrial Science, University of Tokyo, 4-6-1 Komaba, Meguro-ku, Tokyo 153-8505 Japan

**Keywords:** Optical sensors, Electrical and electronic engineering

## Abstract

Terahertz (THz) detectors using MEMS resonators have attracted great interests owing to their high sensitivity, rapid response, and room-temperature operation capability. For easy integration with CMOS circuits, silicon (Si) based MEMS detectors are highly desirable. Here we report an uncooled THz bolometer using doubly-clamped Si on insulator (SOI) MEMS beam resonator with piezoresistive readout. When external heat is applied to the MEMS beam, the resonance frequency shifts owing to the thermal strain in the beam, demonstrating a thermal responsivity up to 149 W^−1^. SOI MEMS resonators exhibit a thermal response time of about 88 μs, which is over 3 times faster than that of GaAs MEMS detectors. Furthermore, electrical readout of the MEMS vibrations is achieved by using the piezoresistive effect of Si, offering a low frequency noise density of 2.7 mHz/√Hz, and subsequently a noise equivalent power (NEP) of about 36 pW/√Hz for the current devices. Optical measurement using a FTIR spectrometer shows that SOI MEMS bolometers has a broadband THz response across 1–10 THz range. These results demonstrate that SOI MEMS bolometer features fast response and high sensitivity, while also being compact, broadband, and CMOS-compatible, highlighting its strong potential for advanced THz spectroscopy and imaging applications.

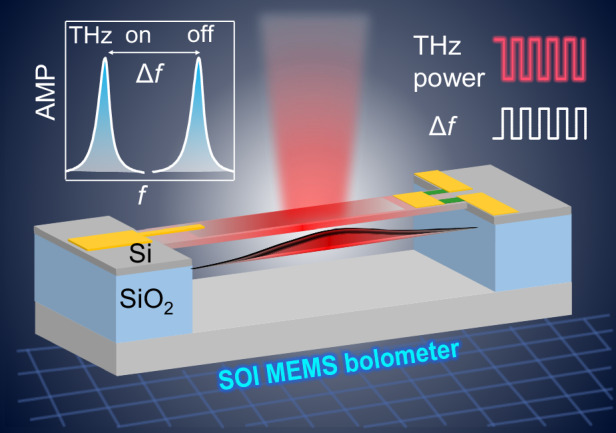

## Introduction

Terahertz (THz) detectors are essential components for applications of THz technology^1^. Detectors using microelectromechanical-system (MEMS) resonators^2–4^ have attracted considerable interests owing to their high sensitivity, rapid response, and room-temperature operation capability. These detectors^5^ typically consist of a MEMS beam or cantilever that acts as a resonant element, detecting incident light by observing changes in their resonance frequencies or oscillation amplitudes. A number of studies have demonstrated that MEMS/NEMS resonators in various geometries, such as membrane^6–13^, cantilever^14–16^, drum^17^, or trampoline^18–22^, can serve as sensitive and fast THz or infrared sensors, with a trade-off between the sensitivity and response time. Our previous work^7,8^ demonstrated a THz bolometer using a GaAs doubly clamped MEMS beam resonator, which utilizes the piezoelectric properties of GaAs for mechanical oscillation detection. This device achieved a noise equivalent power (NEP) of ~90 pW/√Hz, approaching the fundamental phonon limit, and an operation speed of several kHz, which significantly outperforms conventional thermal sensors like pyroelectric sensors and VO_X_ bolometers^23^.

For seamless integration with CMOS circuits to achieve low-cost, large-scale production of detector arrays, CMOS-compatible silicon (Si) based MEMS detectors are highly desirable^24^. Previous work has reported the use of a single crystal Si-on-insulator (SOI) MEMS resonator structure for realizing uncooled THz thermal detectors^14^. This device, utilizing a U-shaped SOI cantilever with two aluminum half-wave dipole antennas, detects incident THz waves through cantilever deformation and achieves an NEP of 20 nW/√Hz and an excellent bandwidth of 150 kHz. To achieve higher detection sensitivity, the doubly clamped beam structure is promising, as its resonance frequency is highly sensitive to internal thermal strain. Moreover, previous work utilized an optical readout scheme for Si cantilever deformation, which is challenging to be integrated into compact MEMS detectors. For low-cost and compact THz sensing applications, the all-electrical driving and readout method is necessary for MEMS bolometers.

We have developed uncooled, broadband, sensitive and fast THz bolometers using SOI MEMS beam resonators with piezoresistive readout. Doubly-clamped MEMS beam resonators of various sizes were fabricated with a high-resistivity SOI wafer. When external heat is applied to the MEMS beam, the resonance frequency shifts owing to the thermal strain in the beam, demonstrating a thermal responsivity up to 149 W^−1^. SOI MEMS resonators exhibit a thermal response time of about 88 μs for a 200-μm-long MEMS beam, which is over 3 times faster than that of GaAs MEMS detectors. Furthermore, electrical readout of the MEMS vibrations is achieved by using the piezo-resistivity of Si. The piezoresistive readout gives a high signal-to-noise ratio comparable with optical detection scheme using a laser Doppler vibrometer (LDV), offering a low frequency noise density of 2.7 mHz/√Hz, and subsequently a noise equivalent power (NEP) of about 36 pW/√Hz for the present devices. THz response of the SOI MEMS bolometer was measured by irradiating a NiCr THz absorber placed at surface of the MEMS beam with a blackbody THz source from a FTIR spectrometer. The preliminary results show that SOI MEMS bolometers have a broadband THz response across the 1–10 THz range, highlighting its potential for broadband THz spectroscopy applications.

## Thermal response of the SOI MEMS resonator

**Fig. 1:**
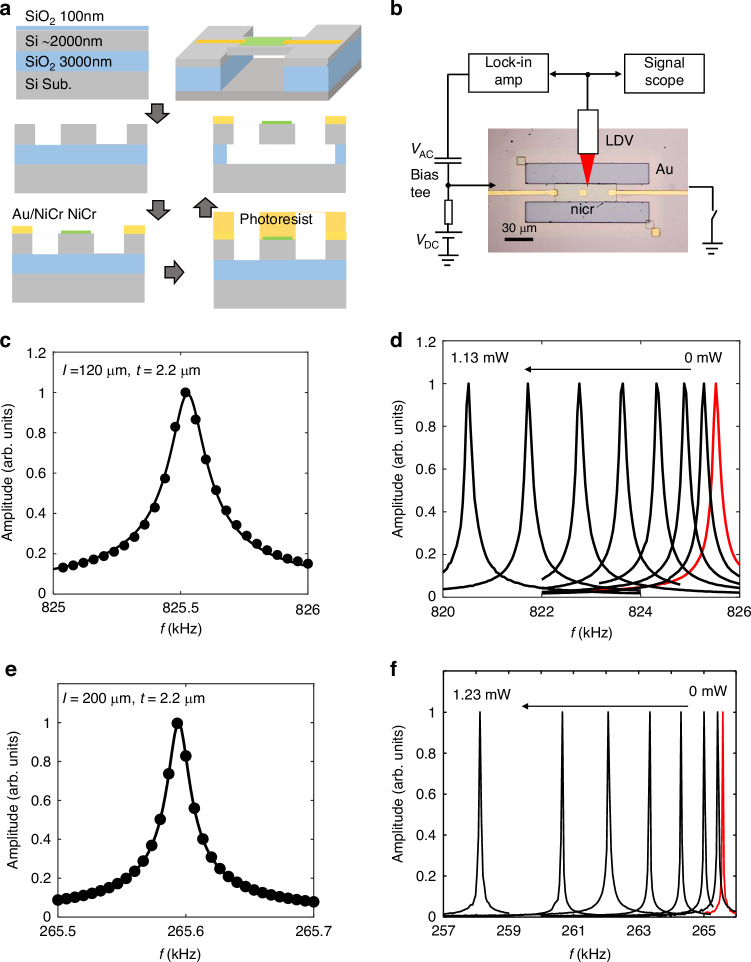
Fabrication and characterization of SOI MEMS resonators. **a** Fabrication process of the SOI MEMS resonator for thermal response measurement. **b** A microscopic image of a fabricated SOI MEMS resonator with the schematic measurement setup. **c** Oscillation spectrum of sample A (*V*_AC_ = 100 mV) with dimensions of 120 μm (*l*) × 30 μm (*w*) × 2.2 μm (*t*). **d** Oscillation spectra of sample A at various heating powers. **e** Oscillation spectrum of sample B (*V*_AC_ = 100 mV) with dimensions of 200 μm(*l*) × 30 μm(*w*) × 2.2 μm(*t*). **f** Oscillation spectra of sample B at various heating powers

We have fabricated MEMS beams of various sizes to study their thermal response. Figure 1a shows the fabrication process and the schematic structure of the MEMS beam. We used a (100)-oriented 2-μm-Si/3-μm-SiO_2_/450-μm-Si wafer with a resistivity >2000 Ω·cm for device fabrication. The thickness of the top Si layer has a fluctuation of ±0.5 μm, and can be reduced by etching process. Two etching windows were formed by tetramethylammonium hydroxide (TMAH) etching of the Si layer with a 100-nm-thick thermal SiO_2_ as a hard mask. Then, a 20-nm-thick nichrome (NiCr) layer was deposited at the center of the beam structure, which functions as a broadband THz absorber, a local heater, and an electrostatic driving electrode for the MEMS beam. Additionally, NiCr (5 nm)/Au (100 nm) electrodes were formed to contact the NiCr heater. Finally, the SiO_2_ beneath the MEMS beam was selectively etched using a buffered hydrofluoric acid (BHF) based etching solution to release the MEMS beam, with the device surface protected using photoresist.

Figure 1b shows a microscope image of a fabricated SOI MEMS beam, as well as the schematic measurement setup for measuring the thermal response. An AC voltage (*V*_AC_ = 100 mV) is applied to the NiCr electrode, with its ground electrode disconnected, ensuring that only electrostatic force is applied without heating the MEMS beam. The measured oscillation spectrum for a sample (sample A) with dimensions of 120 μm(*l*) × 30 μm(*w*) × 2.2 μm(*t*) is shown in Fig. 1c, where *l*, *w* and *t* express the length, width and thickness of the beam. The resonance frequency was observed at *f*_0_ = 825.5 kHz, with a quality(Q)-factor of ~6300. Figure 1d shows the resonance frequency for another sample (sample B) with dimensions of 200 μm(*l*) × 30 μm(*w*) × 2.2 μm(*t*), where *f*_0_ was reduced to ~265 kHz, approximately fulfill the relation that *f*_0_∝*t*/*l*^2^. Notably, this sample exhibited a high Q-factor of approximately 1.6 × 10^4^, which is advantageous for low-noise sensing applications.

To measure the thermal response of the SOI MEMS resonator, a DC voltage (*V*_DC_) is applied to the NiCr heater together with *V*_AC_ through a bias tee circuit, and the ground electrode is connected to allow current flow through the NiCr heater. Here, we keep *V*_DC_ >> V_AC_, so that the generated heat$\,P\approx \frac{{{V}_{{\rm{DC}}}}^{2}}{{R}_{{\rm{NiCr}}}}$. A thermal strain is applied to the MEMS beam, which effectively reduces the tension of the MEMS beam and subsequently reduces the resonance frequency. Figure 1d, f shows the oscillation spectra of the two samples measured at various *P*. As seen, the resonance frequency (*f*_p_) shifts to the lower frequency side as the heating power increases, which is similar to the GaAs MEMS beams as we previously reported^7,8^.

**Fig. 2:**
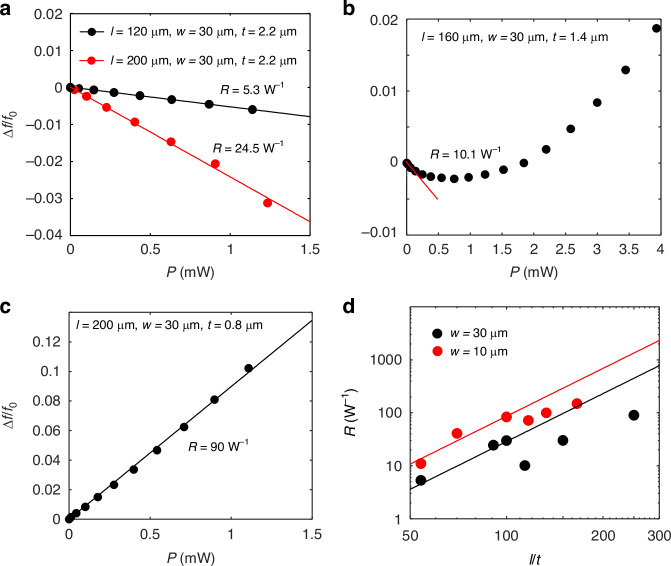
Thermal responsivity of MEMS resonators. **a** Normalized resonance frequency shift (∆*f*/*f*_0_) as a function of the input heat power *P* for sample A (black dots) and sample B (red dots). The black and red lines show the linear fitting of ∆*f*/*f*_0_ as a function of *P*. **b** ∆*f*/*f*_0_ as a function of *P* for a MEMS beam of 160 μm(*l*) × 30 μm(*w*) × 1.4 μm(*t*). **c** ∆*f*/*f*_0_ as a function of *P* for a MEMS beam of 200 μm(*l*) × 30 μm(*w*) × 0.8 μm(*t*). **d** Responsivities as a function of *l*/*t*. The black and red dots plot the data for the samples with *w* = 30 μm and 10 μm respectively; the black and red curves plots the expected *R* calculated from the cubic relation *R*∝(*l*/*t*)^3^ for the MEMS beams with *w* = 30 μm and 10 μm, respectively

The black and red curves in Fig. 2a show the normalized resonance frequency shift ($\frac{\Delta f}{{f}_{0}}=\frac{{f}_{{\rm{p}}}-{f}_{0}}{{f}_{0}}$) as a function of the input heat power *P* for sample A and sample B, obtained from the oscillation spectra shown in Fig. 1d, f, respectively. As seen, both frequency shifts exhibit a good linear relationship. Here we define the thermal responsivity as^8^

1 $$R=\frac{|\Delta f|}{P\times {f}_{0}}$$

Note that it is the frequency shift per unit input power normalized by its intrinsic resonance frequency, thus has a unit of [W^−1^]. As for the doubly clamped beam with no initial internal strain, the thermal responsivity *R* is proportional to the cube of the ratio of beam length (*l*) and thickness (*t*), i.e., *R*∝${\frac{1}{w}(\frac{l}{t})}^{3}$. Thus a large aspect ratio (*l*/*t*) and a small beam width (*w*) would significantly improves the thermal responsivity. With a linear fitting to $\frac{|\Delta f|}{{f}_{0}}$ as a function of *P*, we obtain *R* = ~ 5.3 W^−1^ for sample A (*l*/*t* = ~54.5) by the slope of the black fitting curve shown in Fig. 2a. In comparison, sample B with *l*/*t* = ~91 gives *R* = ~ 24.5 W^−1^ by the slope of the red fitting curve in Fig. 2a, reaching a nice agreement on the cubic relation.

As *l*/*t* exceeds 100, however, a degradation in *R* is observed. Figure 2b shows the normalized resonance frequency shift (∆*f*/*f*_0_) as a function of the input heat power *P*, measured for a MEMS beam with *l*/*t* = 160 μm/1.4 μm = ~114, which only gives a small *R* = ~ 10 W^−1^ when *P* < ~ 0.2 mW. With a further increase in *P*, the resonance frequency levels off and then start to shift to the high-frequency side, indicating that the MEMS beam enters the buckling region (*P* > 1.5 mW)^25–27^. This behavior suggests that there is a residual compressive strain in the MEMS beam, which significantly modifies the thermal response of the MEMS resonator at high *l*/t values. With a further increase in *l*/*t*, the MEMS beam enters the buckling region even before applying any heat. Figure 2c shows the normalized resonance frequency shift (∆*f*/*f*_0_) as a function of *P*, measured for a MEMS beam with *l*/*t* = 200 μm/0.8 μm = ~250. As seen, the resonance shows a blue shift as *P* increases, giving an enhanced *R* = ~ 90 W^−1^.

Furthermore, we have reduced the beam width (*w*) from 30 μm to 10 μm to improve the responsivity. The resonance spectra and thermal response for these 10-μm-width samples can be found in the supplementary note 1. Figure 2d summarizes the responsivities obtained from samples with different aspect ratios and widths. The black and red dots plot the data for the samples with *w* = 30 μm and *w* = 10 μm, respectively. The black and red curves plots the expected *R* calculated from the cubic relation *R*∝(*l*/*t*)^3^ for the MEMS beams with *w* = 30 μm and 10 μm respectively. As shown, beams with a smaller width (10 μm) generally exhibit higher responsivity due to the reduced thermal conductance. The highest responsivity, *R* = ~ 149 W^−1^, is achieved in a MEMS beam with dimensions 200 μm(*l*) × 10 μm(*w*) × 1.2 μm(*t*) and a *f*_0_ = 497.1 kHz. However, the experimental *R* at large aspect ratio is notably lower than the theoretical prediction. This discrepancy suggests that residual internal strain significantly affects the thermal sensitivity, and reducing the internal strain would be an effective way to improve the thermal responsivity.

The origin of this residual strain is not yet fully understood, but it is likely attributed to the mismatch in thermal expansion coefficients between Si (~2.6 ppm/K) and SiO_2_ (~0.5 ppm/K)^28^_._ Multiple annealing processes in the device fabrication, such as wafer bonding, thermal oxidation, and dopant diffusion, can cumulatively induce and alter stress within the MEMS structure. Nonetheless, it has been reported that the annealing temperature, alternative sacrificial or structural layer materials can be used to control such stress^29^, which would be useful for improving the thermal sensitivity with high aspect-ratio beams.

## Thermal response time of the MEMS beam

**Fig. 3:**
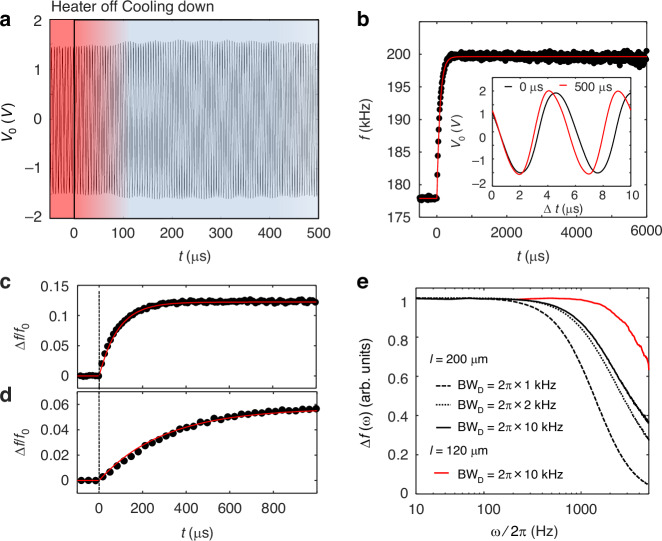
Thermal response dynamics of SOI MEMS resonators. **a** Time trace of the vibration signal when the heating voltage is switched off for a SOI MEMS resonator with a geometry of 200 μm(*l*) × 30 μm(*w*) × 2.0 μm(*t*). **b** The estimated vibration frequency as a function of time. Quick frequency shift is observed as the heating voltage is switched off near *t* = 0 μs. The inset shows a blow-up of the vibration signals started at 0 μs (black curve) and 500 μs (red curve). **c** Normalized frequency shift as a function of time for the SOI MEMS resonator, showing a thermal time constant of ~88 μs. **d** Normalized frequency shift as a function of time for a reference GaAs MEMS resonator with a geometry of 200 μm(*l*) × 30 μm(*w*) × 1.4 μm(*t*), showing a thermal time constant of ~296 μs. **e** Normalized frequency shift as a function of the heat modulation frequency. Black dotted, dashed and solid curves plot the measurement results for the SOI beam with *l* = 200 μm at BW_D_ = 1 kHz, 2 kHz and 10 kHz, respectively. Red curve plot the result for the SOI beam with *l* = 120 μm at BW_D_ = 10 kHz

The thermal decay time (*τ*) of the device is determined by the effective heat capacity(*C*_T_) and thermal conductance (*G*_T_) of the MEMS beam, i.e, *τ* ≡ *C*_T_ /*G*_T_. With given material properties and beam shapes, *τ* is determined by the length of the MEMS beam. This is because the change in width or thickness of the MEMS beam will modulates *C*_T_ and *G*_T_ in the same scale, thus maintains the same *τ*. We have studied the thermal decay process using a 200-μm-long MEMS beam. We heated up the MEMS beam by applying a DC voltage to the NiCr heater, and drove the beam at its resonance frequency. Then the DC voltage was switched off, and the resonance frequency shifts to the higher frequency as the MEMS beam cools down. Since the MEMS beam has a large oscillation amplitude, it is in a free-running mode with gradually reducing oscillation amplitude. The time trace of the vibration signal before and after the heat-off operation (*t* = 0 μs) is plotted in Fig. 3a, and a blow up of the vibration signal is shown in the inset of Fig. 3b. As seen, the vibration at 0–10 μs shows a notably longer period than the vibration at 500–510 μs, which is attributed to the cooling of the MEMS beam.

The vibration frequency is then calculated from the period of the vibration, which is plotted in Fig. 3b, showing the thermal decay process in the MEMS beam. As seen, when heater is switched off (*t* = 0 μs), the resonance frequency shifts to a higher frequency quickly, indicating that the thermal decay process is rather fast. Figure 3c shows a normalized frequency shift as a function of time. By fitting the frequency shift curve, we have estimated that the thermal time constant *τ*_SOI_ = ~88 μs in this sample. The fitting result is plotted as the red curve in Fig. 3c. Figure 3d shows the frequency transition behavior for a reference GaAs doubly clamped MEMS beam resonator, with the same beam length (*l* = 200 μm), giving a thermal decay time *τ*_GaAs_ = ~296 μs. We therefore can conclude that SOI MEMS resonators are over 3 times faster than the GaAs MEMS resonators.

The thermal decay time determines the intrinsic thermal modulation bandwidth of the bolometer, given by BW_T_ $=\frac{1}{{2\pi \tau }_{{\rm{SOI}}}}$ ≈ 1.8 kHz for the 200-μm-long SOI MEMS beam. This thermal bandwidth, together with the demodulation bandwidth (BW_D_) of the electrical circuit used to track ∆*f*, jointly determine the overall modulation bandwidth of the detector (BW_-3dB_). In our measurement, a phase lock loop (PLL) was employed to continuously track ∆*f*^7^. Figure 3e shows the measured normalized ∆*f*(*ω*) as a function of the modulation frequency(*ω*/2π) of input heat. The black dashed and dotted lines correspond to cases where the demodulation bandwidth is limited (BW_D_ = 1 kHz, 2 kHz), yielding BW_-3dB_ of approximately 1 kHz and 1.6 kHz, respectively. When the demodulation bandwidth is sufficiently large (e.g., BW_D_ = 10 kHz), the system can reach the intrinsic modulation limit, as shown by the black solid curve in Fig. 3e with BW_−3dB_ ≈ 1.8 kHz.

Importantly, since *τ*_SOI_∝1/*l*^2^, a shorter beam length results in a significantly reduced thermal time constant, enabling a larger modulation bandwidth. This is demonstrated by the red curve in Fig. 3e, which is the measured ∆*f*(*ω*) for a 120-μm-long beam. The measured BW_-3dB_ = 4.7 kHz, corresponding to a thermal response time of 34 μs. These results demonstrate that SOI MEMS bolometers can operate at modulation frequencies of several kilohertz, making them highly suitable for high-speed THz detection applications.

## SOI MEMS resonator with piezoresistive readout

**Fig. 4:**
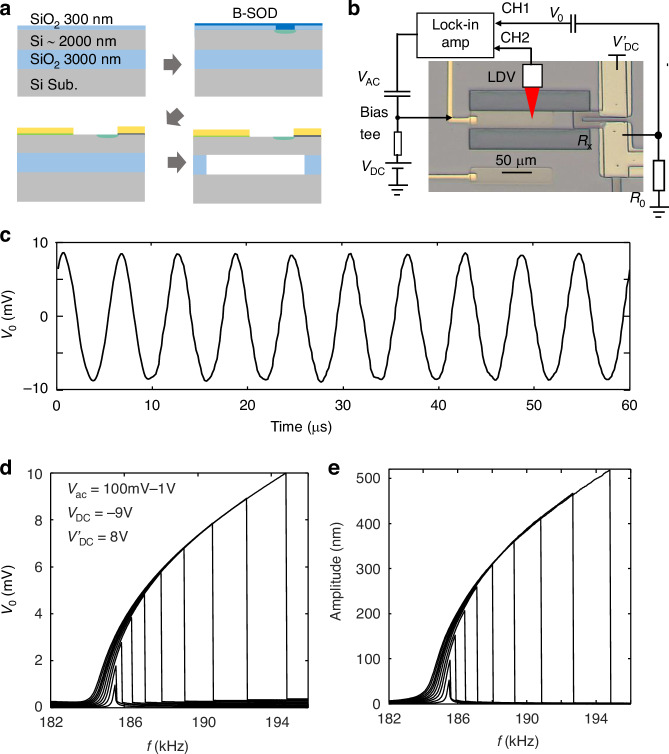
Fabrication and characterization of SOI MEMS resonator with piezoresistive readout. **a** Fabrication process of the SOI MEMS resonator with a piezoresistive readout. **b** A microscopic image of a fabricated MEMS resonator and the schematic measurement setup. *R*_x_ shows the piezoresistive part, and *R*_x_ shows a reference resistor. **c** Measured RF output of the piezoresistive readout (*V*_0_) when the MEMS resonator is driving into resonance. **d** Measured oscillation spectra of the MEMS beam with the piezoresistive readout at various driving conditions. **e** Measured oscillation spectra of the MEMS beam with LDV

Piezoresistive^30^ readout offers significant advantages in scaling when reducing device size, compared to capacitance-based readout methods^31–33^. Piezoresistive readout utilizes the piezo-resistivity of a doped Si layer to detect vibrations of MEMS beams. For out-of-plane modes typically used in MEMS thermal sensors, it is essential to control the doping layer only at the surface of the beam. The fabrication process is based on a spin-on-doping (SOD) process, as schematically illustrated in Fig. 4a. We deposited a 300-nm-thick SiO_2_ layer on the surface of the SOI wafer via chemical vapor deposition (CVD), which serves as a hard mask for doping^34^. The doping was performed by using a boron spin-on coating solution^35^. A 10-min rapid diffusion treatment was performed in nitrogen atmosphere at 1000 °C, ensuring that the carriers only exist at the surface of the MEMS beam. Cr(20 nm)/Au(100 nm) electrodes were formed on the doped layer to establish ohmic contacts to the piezoresistor. Additionally, a thin NiCr layer of 20 nm was formed on the MEMS beam to form an electrostatic driving electrode, which also serves as an absorber for the incident THz waves. Finally, SiO_2_ beneath the MEMS beam was removed to release the suspended MEMS beam.

A microscope image of the fabricated MEMS resonator is shown in Fig. 4b. The resistance of the piezoresistor was measured to be about 610 Ω, with possible variations of ±20% due to fabrication inconsistencies. The piezoresistor (*R*_x_) was connected with a reference resistor (*R*_0_), and a DC voltage (*V’*_DC_) was applied to the circuit, as schematically illustrated in Fig. 4b. When *R*_x_ changes due to the piezoresistive effect, the output voltage (*V*_0_) is given by the equation:

2 $${V}_{{\rm{o}}}={V^{\prime} }_{{\rm{DC}}}\times \frac{{R}_{x}}{{R}_{0}}\frac{\Delta {{R}_{x}/R}_{x}}{{(1+{R}_{x}/{R}_{0})}^{2}}$$

Since the MEMS resonator vibrates in the sub-MHz to MHz range, *V*_0_ can be regarded as an RF signal converted from mechanical vibrations. We used a reference resistor *R*_0_ ≈ *R*_x_ = 610 Ω and *V*’_DC_ = 8 V for the measurement. An ac voltage (*V*_AC_) with a DC offset *V*_DC_ = − 9 V to applied to the driving electrode to electrostatically excite the mechanical vibrations. Figure 4c shows the measured *V*_o_ when the MEMS beam is excited into vibrations at ~190 kHz at *V*_AC_ = 500 mV. As seen, a clear RF signal of an peak-to-peak amplitude of exceeding 10 mV was observed, attributed to the piezoresistive effect of *R*_x_.

To investigate the displacement sensitivity, we used the lock-in amplifier to simultaneously detect the oscillation spectra of the MEMS beam from the piezoresistive readout and a LDV, as schematically shown in Fig. 4b. Figure 4d shows the oscillation spectra of the MEMS resonator at various driving voltages using the piezoresistive readout circuit. As *V*_AC_ increases from 100 mV to 1 V, the oscillation amplitude, i.e., the RMS amplitude of *V*_o_, increases from 1 mV to 10 mV. The resonance frequency increases at higher oscillation amplitudes due to the mechanical nonlinearity of the MEMS beam. Figure 4e presents the oscillation spectra measured by the LDV under the same driving conditions, and y-axis shows the oscillation amplitudes, i.e., RMS amplitude of the vertical deflection measured at the center of the MEMS beam. From Fig. 4d, e, the piezoresistive readout reaches a voltage sensitivity of *v*_s_ = 19.4 μV/nm, and achieves a high signal-to-noise ratio that is comparable to optical vibration measurement techniques.

## Noise behavior of SOI MEMS resonator

**Fig. 5:**
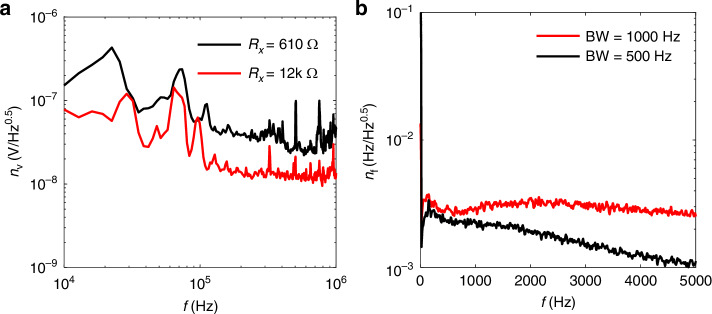
Noise characterization of piezoresistive readout. **a** Voltage Noise density spectra of the piezoresistive readout with *V*’_DC_ = 8 V for the condition *R*_x_ = ~610 Ω, *R*_x_ = ~12 kΩ, respectively. In each measurement, *R*_0_ = *R*_x_. **b** Frequency noise density of the MEMS resonator with piezoresistive readout. The MEMS beam is driven with *V*_DC_ = − 9 V, *V*_AC_ = 1 V, *V*’_DC_ = 8 V at its resonance frequency by using a phase-locked loop, with a demodulation frequency of 500 Hz (black curve) and 1000 Hz (red curve)

The black curve in Fig. 5a shows the voltage noise spectrum of the piezoresistive readout. Since the piezoresistor has a low resistance of ~600 Ω, the Johnson-Nyquist noise is rather small. The noise spectrum reveals a clear 1/*f* noise, which may be due to the random trapping and detrapping process of charge carriers at the dopant sites. Temperature rise caused by the electro-thermal effect of the piezoresistor may also contributes to the 1/*f* noise. At the operating frequency (~200 kHz) of the MEMS resonator, the noise density *n*_v_ = ~40 nV/√Hz.

To improve the noise performance, we have performed a surface etching process to the piezoresistor by using reaction ionic etching (RIE). Experimental detail is shown in the Supplementary note 2. After the etching, the resistance increases from ~600 Ω to ~12 kΩ. Keeping the same bias condition and *R*_0_ = *R*_x_, we have measured the noise spectrum of the piezoresistive readout, the result is shown as the red curve in Fig. 5a. As seen, the noise density is significantly reduced. At the resonance frequency (~200 kHz), the noise density becomes 14 nV/√Hz, corresponding to a displacement sensitivity of *n*_v_/*v*_s_ = ~0.7 pm/√Hz. The displacement sensitivity is comparable with the optical readout schemes such as the LDV, or balance detector^14^. In addition, the power consumption for the piezoresistor decreases from ~52 mW to ~2.7 mW.

Figure 5b shows the measured frequency noise densities with the PLL demodulation BW of 1 kHz (red curve) and 500 Hz (black curve), respectively, where the PLL is used to maintain the self-oscillation of the MEMS resonator. PLL demodulation BW of 1 kHz and at a modulation frequency of 600 Hz and *V*_o_ = 10 mV(RMS), the frequency noise density reaches the minimum of *n*_f_ = ~2.7 mHz/√Hz, which is similar to the lowest *n*_f_ we have observed for the GaAs MEMS bolometer^7^. The noise equivalent power(NEP) of the device is calculated by NEP ≡ *n*_f_ /(*f*_0_×*R*). Taking *n*_f_ = 2.7 mHz/√Hz, *f*_0_ and *R* for the samples with different geometries, we have estimated that the sample 200 μm(*l*) × 30 μm(*w*) × 0.8 μm(*t*) has an NEP of 178 pW/√Hz, and the sample 200 μm(*l*) × 10 μm(*w*) × 1.2 μm(*t*) has an NEP of as low as 36 pW/√Hz. This NEP is comparable with the recent reported near-infrared SOI MEMS detector using the differential output of dual-mode coupled resoantors^36^. Furthermore, our device features a shorter thermal response time and a more straightforward operation principle.

Furthermore, it is valuable to know the extreme detection sensitivity of the device. The thermal fluctuation noise represents the fundamental sensitivity limit given by the random transfer of energy between the MEMS beam and the thermal reservation. It can be calculated by the thermal conductance (*G*_T_) of the MEMS beam, as, NEP_TF_ = (4*k*_B_*T*^2^*G*_T_)^0.5^ ≈ 17.7 pW/√Hz at *T* = 300 K, for a 200 μm(*l*) × 10 μm(*w*) × 1.2 μm(*t*) Si beam. The present NEP = 36 pW/√Hz, which is about 2 times of NEP_TF_, indicating that NEP has been approaching the fundamental phonon limit of the device.

## Detection of broadband THz radiation with SOI MEMS bolometer

**Fig. 6:**
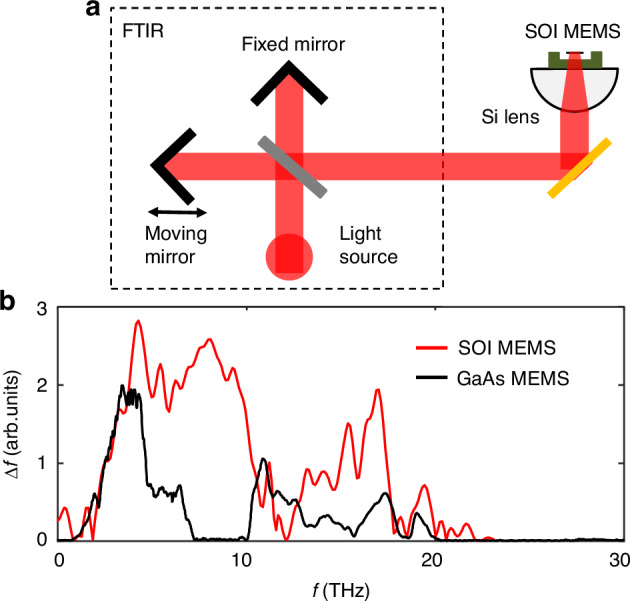
Optical measurement setup and THz response comparison. **a** Schematic optical measurement setup for the THz irradiation measurement. **b** The normalized THz response spectra for a SOI MEMS resonator (red curve) and a GaAs MEMS resonator (black curve)

A key advantage of the SOI MEMS bolometer is its broadband spectral response. In contrast, the GaAs MEMS bolometer we previously reported exhibits spectral limitations due to the polar nature of GaAs, which causes complex interactions between THz waves and acoustic/optical phonons. This results in reduced sensitivity at 5–7 THz (acoustic phonon band) and zero sensitivity at 7–10 THz (TO and LO phonon bands)^37^. On the other hand, Si, being a non-polar material, is expected to deliver a uniform spectral response across the THz range, offering a significant advantage for broadband THz sensing applications. To verify this, we performed THz irradiation measurements on both SOI and GaAs MEMS bolometers. The light source is a blackbody source at approximately 1000 °C, provided by an FTIR spectrometer (JASCO FT-6300), and the optical setup is illustrated schematically in Fig. 6a. The light beam was split into two beams, with one beam’s optical path modulated by a moving mirror to generate interference. The light interference is measured by the MEMS bolometer, with Fourier transform applied to obtain the THz response spectra. Furthermore, because the light source is not a point source, focusing the light onto the small absorption area of the MEMS bolometer is challenging. To address this, the MEMS bolometer is mounted on a hyper-hemisphere Si lens to enhance light collection efficiency.

The NiCr absorber has a sheet resistivity of ~100 Ω/sq, providing a broadband absorption coefficient of ~20% to the incident THz wave^38^. However, since both the beam splitter and optical windows of the FTIR has a finite spectral bandwidth, the irradiated light vanishes at ~20 THz. The measurement results are shown in Fig. 6b. The red curve in Fig. 6b represents the frequency response (∆*f*) spectrum of the SOI MEMS bolometer, measured with a spectral resolution of 16 cm^−1^(~0.5 THz). For comparison, the black curve in Fig. 6b shows the frequency response spectrum of the GaAs MEMS bolometer measured with the same FTIR spectrometer. The reference GaAs MEMS bolometer has a geometry of 400 μm(*l*) × 30 μm(*w*) × 1.2 μm(*t*). As observed, there is a zero-sensitivity frequency band at 7–10 THz, which is owing to the light-phonon interactions in GaAs material. In contrast, SOI MEMS bolometer shows good response in this band, indicating that SOI MEMS bolometer is better suited for broadband THz sensing applications.

## Conclusion and discussion

In summary, we have developed an uncooled, broadband, highly sensitive and fast THz bolometer based on a SOI MEMS beam resonator with piezoresistive readout. Notably, the device is fabricated using commercial SOI wafers and a fully CMOS-compatible Si process, making it low-cost, scalable, and ideal for monolithic integration with other Si-based electronic and photonic components.

**Fig. 7:**
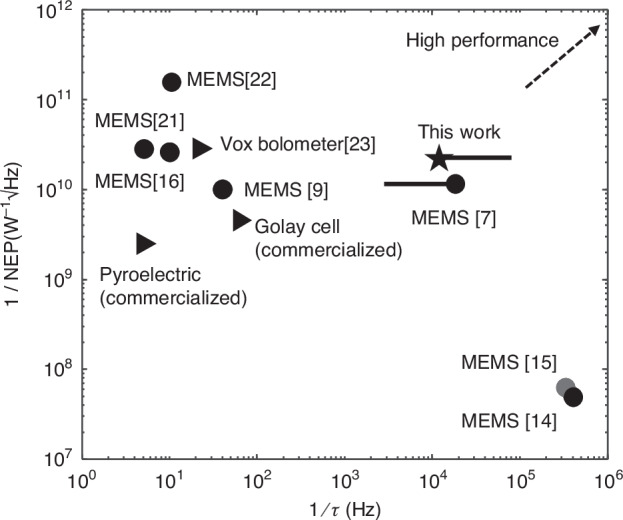
Comparison of the sensitivity (1/NEP) and thermal response speed (1/τ) of SOI MEMS bolometer against state-of-the-art uncooled THz bolometers based on MEMS resonators, as well as widely used uncooled thermal detectors such as pyroelectric detectors, Golay cells, and VOx bolometers

To benchmark the performance, Fig. 7 presents a comparison of the sensitivity (1/NEP) and thermal response speed (1/*τ*) of our SOI MEMS bolometer against state-of-the-art uncooled THz bolometers based on MEMS resonators, as well as widely used uncooled thermal detectors such as pyroelectric detectors, Golay cells, and VOx bolometers. The SOI MEMS bolometer achieves a modulation bandwidth of several kilohertz, ~100–1000 times faster than traditional thermal detectors while maintaining competitive sensitivity. The overall performance, in terms of $\frac{1}{{\rm{NEP}}}\cdot \frac{1}{{\rm{\tau }}}$, as shown in Fig. 7, is better or at the same level with other state-of-art THz detectors based on GaAs^7,15^, Si nitride^9,21,22^,or SOI-Al^14^ MEMS resonators, as shown in Fig. 7.

Furthermore, the SOI MEMS bolometer demonstrates a broadband spectral response across the 1–10 THz range. This level of broadband operation is difficult to achieve with GaAs or other compound semiconductor-based MEMS detectors due to strong optical phonon interaction in the material. Overall, our SOI MEMS bolometer provides a compelling solution for high-speed, high-sensitivity, and broadband THz detection, with strong potential for on-chip integration and mass production for spectroscopy and imaging applications.

If the discussion is extended beyond thermal-type THz detectors, THz rectifiers based on Schottky diodes^39–41^ or field-effect transistors (FETs)^42^, also represent promising uncooled THz detection technologies. These rectifiers can achieve ultra-high sensitivity, with reported NEP as low as 0.5–10 pW/√Hz^43^ in the sub-THz range. However, their sensitivity typically drops significantly above ~1.5 THz, limiting their effectiveness for broadband THz sensing applications. In contrast, MEMS bolometers offer a broadband THz spectral response, as demonstrated in Fig. 6, and can be extend to other spectral regions, making them particularly well-suited for THz spectroscopy and imaging applications that require wide frequency coverage.

THz detectors based on two-dimensional (2D) materials^44,45^, including but not limited to graphene, MoS_2_, or PdSe_2_, have demonstrated great potential for uncooled THz sensing. These detectors leverage various mechanisms such as the photothermal effect^46^, bolometric effect^47^, and rectifying effect^48^, demonstrating high sensitivity as well as ultrafast response time, making them highly attractive for ultrafast THz applications. However, MEMS bolometers offer key advantages in terms of fabrication maturity, CMOS process compatibility, and high device reproducibility, where 2D material-based detectors still face substantial challenges.

## Outlook

To advance the SOI MEMS bolometer toward practical applications, real-world performance validation is essential. Since the device operation relies on the mechanical properties of crystalline Si, highly stable performance can be expected. Demonstrating fast THz spectroscopy and imaging in practical scenarios would be a crucial next step. Currently, the device consumes power on the order of milliwatts, primarily due to the operating current in the piezoresistive readout circuit. While this is acceptable for a single detector, it is not sufficiently low for integration into large-scale detector arrays. One potential approach to reduce power consumption is to increase the resistance of the piezoresistor. For example, using a 100 kΩ piezoresistor with a 10 μA detection current would reduce power consumption to ~10 μW. However, increasing the resistance also raises the Johnson–Nyquist noise, requiring careful optimization to balance power consumption and readout sensitivity.

Furthermore, MEMS bolometers generally exhibit a trade-off between sensitivity and response speed; however, reducing device dimensions can help mitigate this limitation. In our devices, 10-μm-wide beams show improved responsivity and NEP over 30-μm-wide beams, without sacrificing thermal response time. This suggests that further scaling to sub-micrometer dimensions could enable NEMS bolometers with much higher sensitivity, potentially reaching NEP levels of a few pW/√Hz or even sub-pW/√Hz^49^. However, reducing the device dimensions also brings challenging. Most existing NEMS devices rely on optical readout^50^, which, while sensitive, poses challenges for compact integration. In contrast, the piezoresistive readout demonstrated here offers good scalability and CMOS compatibility, making it more suitable for Si-based integrated NEMS systems. Another remaining challenge is the limited absorption area in NEMS-scale detectors. However, metamaterial-based THz absorbers can provide effective absorption areas based on the wavelength rather than physical size^14,15^, offering a promising route toward compact, fast, and highly sensitive THz NEMS bolometers.

## Supplementary information

Supplementary Notes
