# Supplementary material for: Uncooled, broadband terahertz bolometers using SOI MEMS beam resonators with piezoresistive readout
Source: Microsyst Nanoeng. 2025 Jul 7;11:132. doi: 10.1038/s41378-025-00996-2 (PMC12234989; doi:10.1038/s41378-025-00996-2)
Supplement: Supplementary file 1 — Supplementary Notes [file 41378_2025_996_MOESM1_ESM.pdf]

Uncooled, Broadband Terahertz Bolometers using SOI MEMS Beam Resonators with Piezoresistive Readout: supplementary information

Ya Zhang[1,a)], Kazuki Ebata[1], Mirai Iimori[1], Qian Liu[1], Zihao Zhao[1],Ryotaro Takeuchi[1], Hua Li[2], Kazusuke Maenaka[3], and Kazuhiko Hirakawa[4]

[1]*Institute of Engineering, Tokyo University of Agriculture and Technology, 2-24-16 Koganei-shi, Tokyo, 184-8588, Japan*

[2]*Shanghai Institute of Microsystem and Information Technology, Chinese Academy of Sciences, Shanghai 200050, China*

[3]*Department of Electrical Engineering and Computer Sciences, University of Hyogo, Himeji, Japan*

[4]*Institute of Industrial Science, University of Tokyo, 4-6-1 Komaba, Meguro-ku, Tokyo 153-8505, Japan*

1. Thermal response in 10-µm-wide SOI MEMS beam resonator

To improve thermal sensitivity, we have fabricated MEMS beam resonators with reduced beam widths. The device design and fabrication process are the same as those shown in Fig. 1, except that the beam width is reduced from 30 µm to 10 µm. A threefold improvement in thermal responsivity is expected. Typical measurement results of the fabricated samples are shown in Figs. S1–S3. Data for additional samples are available upon request.

Figure S1(a) shows a microscopic image of the MEMS beam with dimensions of 120 µm (*l*)×10 µm (*w*) ×2.2µm (*t*). Figure S1(b) presents the measured resonance spectra under various heating powers. The measurement procedure is the same as described in the section "Thermal response of the SOI MEMS resonator." As shown, the intrinsic resonance frequency is ~977.5 kHz, which shifts to lower frequencies as the input heating power increases. Figure S1(c) shows the normalized frequency shift as a function of heating power, yielding a thermal responsivity R=11 W^{-1} for this sample.

Figures S2 and S3 show the microscope images and measurement results for MEMS beams with dimensions of 120 µm (*l*)×10 µm (*w*) ×1.2 µm (*t*), and 200 µm (*l*)×10 µm (*w*) ×1.2 µm (*t*), respectively, demonstrating improved thermal responsivities of 84 W^{-1} and 149 W^{-1}.

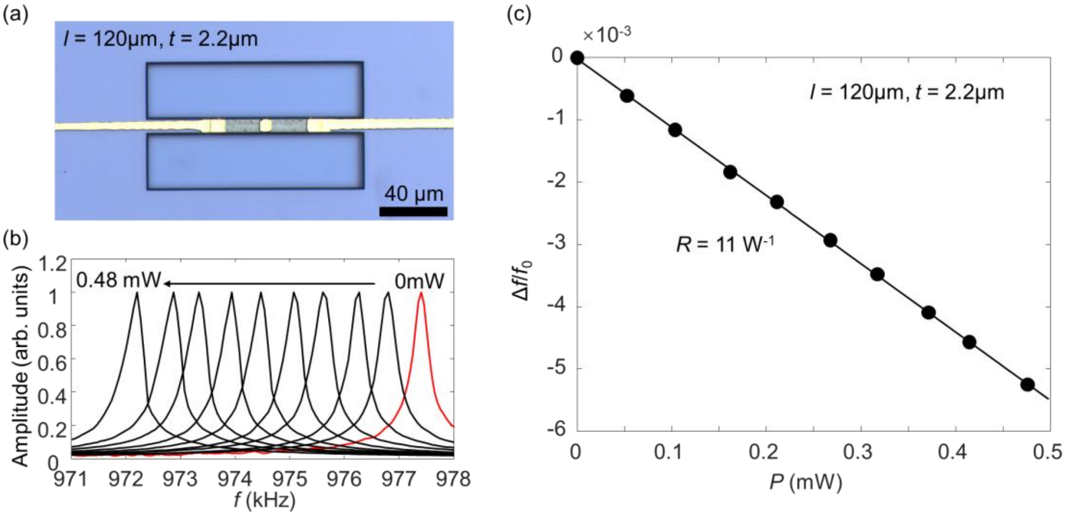

Figure S1 (a) A microscopic image of the MEMS resonator with dimensions of 120 µm (*l*)×10 µm (*w*) ×2.2µm (*t*). (b) Oscillation spectra at various heating powers. (c) Normalized resonance frequency shift ($\Delta f/f_0$) as a function of the input heat power P. The black lines show the linear fitting of $\Delta f/f_0$ as a function of P.

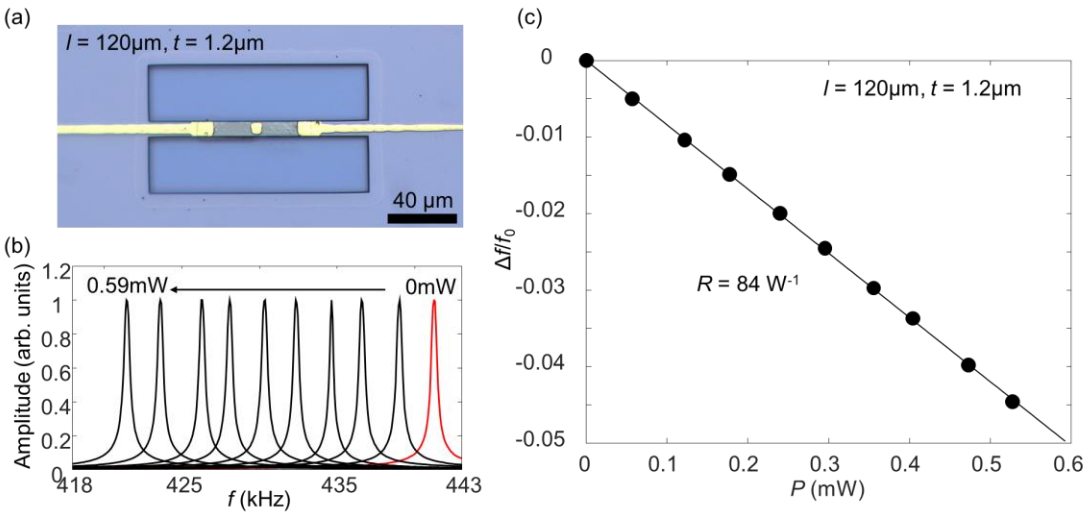

Figure S2 (a) A microscopic image of the fabricated resonator with dimensions of 120 μm (l)×10 μm (w) ×1.2 μm (t). (b) Oscillation spectra at various heating powers. (c) Normalized resonance frequency shift ($\Delta f/f_0$) as a function of the input heat power P. The black lines show the linear fitting of $\Delta f/f_0$ as a function of P.

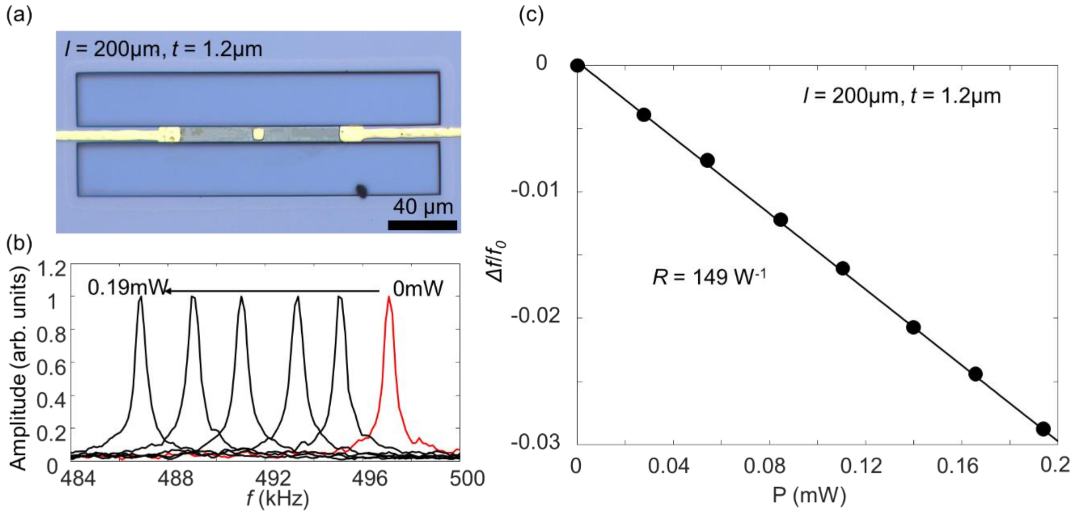

Figure S3 (a) A microscopic image of the fabricated resonator with a dimension of 200 μm (l)×10 μm (w) ×1.2 μm (t). (b) Oscillation spectra at various heating powers. (c) Normalized resonance frequency shift ($\Delta f/f_0$) as a function of the input heat power P. The black lines show the linear fitting of $\Delta f/f_0$ as a function of P.

2. Improvement in the noise performance of the piezoresistive readout

Since the flicker noise is attributed to the carrier trapping-detrapping process and the defects

related processes, we think reducing the impurity density must reduce the flicker noise. With this idea, we have used a reactive ionic etching process to clean and etch the surface of the piezoresistor.

We first investigated the doping density profile with a dummy SOI wafer, which is doped following the same doping process with the sample presented in the manuscript. We used the reaction ionic etching (RIE), with CF_4 gas flow at a RF power of 80 W to etch the surface of the dummy sample, and measured the etching depth with a step meter. Then, we measured the sheet resistance at various etching depths, and the result is shown in Fig.S4(a). The increase in the sheet resistance indicates that the etching process removed the highly doped layer. With the resistance-depth data, we have estimated the carrier concentration as a function of the depth in the Si layer, and the result is shown in Fig. S4(b). As shown, the peak doping density is >10^{19} cm^{-3}, and drops to 10^{18} cm^{-3} at the depth of 250-300 nm.

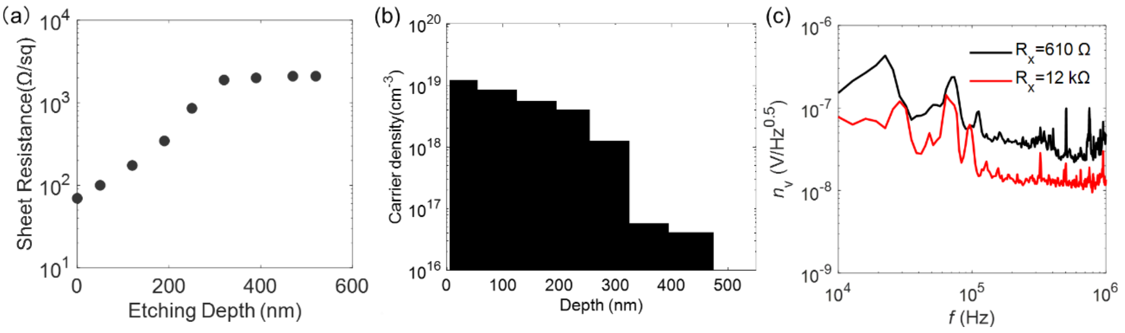

Figure S4 (a) The sheet resistance of the SOD doped silicon reference sample as a function of the etching depth. (b) The calculated carrier density as a function of the depth. (3) Noise density spectra before (red) and after (black) the RIE etching of the piezoresistive resistor.

With this knowledge, we have performed RIE etching to the fabricated SOI MEMS beam to reduce the doping concentration of piezoresistor. The sample was etched by ~280 nm. After the etching process, the resistance of the piezoresistor increases from ~600 Ω to ~12 kΩ. Keeping the same bias condition and adjusting the reference resistor R_0=~ R_x. We have measured the noise spectrum of the piezoresistive readout, and the result is shown as the red curve in Fig. S4(c).The noise density is significantly reduced. At the resonance frequency (~200 kHz), the noise density becomes 14 nV/√Hz, which is reduced by 60% comparing with the sample before etching.
